# Low-Dose Arsenic Compromises the Immune Response to Influenza A Infection *in Vivo*

**DOI:** 10.1289/ehp.0900911

**Published:** 2009-05-20

**Authors:** Courtney D. Kozul, Kenneth H. Ely, Richard I. Enelow, Joshua W. Hamilton

**Affiliations:** 1 Department of Pharmacology and Toxicology, Dartmouth Medical School, Hanover, New Hampshire, USA; 2 Department of Medicine and; 3 Departments of Medicine and Microbiology, Dartmouth Medical School, Lebanon, New Hampshire, USA; 4 Bay Paul Center in Comparative Molecular Biology and Evolution, Marine Biological Laboratory, Woods Hole, Massachusetts, USA

**Keywords:** arsenic, dendritic cells, influenza, innate immune system, mouse lung

## Abstract

**Background:**

Arsenic exposure is a significant worldwide environmental health concern. We recently reported that 5-week exposure to environmentally relevant levels (10 and 100 ppb) of As in drinking water significantly altered components of the innate immune response in mouse lung, which we hypothesize is an important contributor to the increased risk of lung disease in exposed human populations.

**Objectives:**

We investigated the effects of As exposure on respiratory influenza A (H1N1) virus infection, a common and potentially fatal disease.

**Methods:**

In this study, we exposed C57BL/6J mice to 100 ppb As in drinking water for 5 weeks, followed by intranasal inoculation with a sub lethal dose of influenza A/PuertoRico/8/34 (H1N1) virus. Multiple end points were assessed postinfection.

**Results:**

Arsenic was associated with a number of significant changes in response to influenza, including an increase in morbidity and higher pulmonary influenza virus titers on day 7 post-infection. We also found many alterations in the immune response relative to As-unexposed controls, including a decrease in the number of dendritic cells in the mediastinal lymph nodes early in the course of infection.

**Conclusions:**

Our data indicate that chronic As exposure significantly compromises the immune response to infection. Alterations in response to repeated lung infection may also contribute to other chronic illnesses, such as bronchiectasis, which is elevated by As exposure in epidemiology studies.

Chronic exposure to arsenic is a significant worldwide environmental health concern [Agency for Toxic Substances and Disease Registry (ASTDR) 1999; National Research Council 1999]. Contamination of drinking water by natural geologic sources of As is the primary route of exposure. The U.S. Environmental Protection Agency (EPA) standard for drinking water As exposure was recently reduced to 10 ppb (0.13 μM) (U.S. EPA 2001). However, this standard does not cover private wells. In many areas of the United States As is naturally found at levels higher than the federal guidelines, and a signifi cant portion of the population may be drinking excess As chronically (Karagas et al. 2002). This may represent as many as 25 million people in the United States, and worldwide hundreds of millions of people are exposed to levels of As far above 10 ppb. In addition, significant biological effects of As have been observed in cell culture and in animal models at and below the current 10-ppb U.S. EPA standard (Andrew et al. 2007; Kozul et al. 2009; Lantz et al. 2007; Straub et al. 2008).

Chronic exposure to As has been associated with many diseases, including lung, liver, skin, kidney, and bladder cancer; cardiovascular disease; diabetes; and reproductive and developmental defects (Abernathy et al. 1999; National Research Council 1999; Smith et al. 1992; Tapio and Grosche 2006; Watanabe et al. 2003). Multiple mechanisms have been associated with As-induced disease risk, including endocrine disruption, oxidative stress, and alterations in cell signaling and DNA repair (Andrew et al. 2006; Aposhian and Aposhian 2006; Kaltreider et al. 2001; Rossman 2003). However, differences in dose, time, and tissue, as well as coexposures, can result in differing mechanisms and complicate the interpretation of disease risk under varying exposure conditions. Recent reports have indicated that chronic As exposure in human populations results in an increased risk of a variety of lung diseases, including impaired lung function, bronchiectasis, lung cancer, and other respiratory illnesses (Ghosh et al. 2007; Raqib et al. 2009; Smith et al. 2006). The ability of As to increase the risk of lung disease through ingestion, as opposed to inhalation, makes it a unique and intriguing lung toxicant.

Arsenic has been identified as a potent immunomodulatory agent in many experimental models and epidemiologic studies (Andrew et al. 2008; Hernandez-Castro et al. 2009; Lemarie et al. 2006; Nayak et al. 2007; Zhou et al. 2006). We recently showed that chronic low-dose As exposure can profoundly alter the gene and protein expression of many regulators of the innate immune system in a mouse model of exposure (Kozul et al. 2009). We hypothesized that As-induced alterations on the immune system in the lung will lead to a compromised response to a subsequent immune challenge. In the present study, we tested this hypothesis by investigating whether chronic low-dose As exposure could affect the severity of influenza A (H1N1) infection in a murine model. Based on our previous studies, we were specifically interested in the innate immune response to infection, including the migration capability of innate immune cells, such as dendritic cells (DCs). DCs are antigen-presenting cells (APCs) that activate naive T cells, and their functioning is critical to the initiation of a primary immune response. With particular respect to the lung, where there is persistent exposure to environmental pathogens, DCs are constantly sampling the environment, processing antigen, and controlling tolerance, demonstrating their indispensable role in immune regulation.

Respiratory infections with influenza are a significant public health concern and a major cause of morbidity and mortality worldwide (Fauci 2005). It has been estimated that 5–15% of the population will contract influenza infection annually, resulting in > 3–5 million hospitalizations and between 250,000 and 500,000 deaths worldwide (World Health Organization 2009). Identifying risk factors, including environmental exposures, such as As, could have a substantial and immediate impact on public health (Lawrence 2007). Additionally, the impact of As exposure on the potential for a pandemic flu outbreak is of particular concern, considering that many of the geographic areas with confirmed human cases of avian flu or H1N1 (swine) flu are known to include populations that also have significantly elevated As exposures, such as in Southeast Asia and Mexico. The recent outbreak of H1N1 flu in Mexico and across the world has demonstrated the impact a pandemic flu can have on global populations. Age and under lying medical conditions influence susceptibility and severity of infection; however, these factors cannot account for the extreme variability observed in response to influenza infection. Many questions have been raised concerning the increased mortality as a result of H1N1 infection in Mexico compared with relatively mild disease in other infected populations. There is growing evidence that exposure to environmental toxicants, including dioxin and cigarette smoke, can dramatically alter antiviral responses (Burleson et al. 1996; Gualano et al. 2008; Warren et al. 2000). In the present study, we show that chronic low-dose As exposure can profoundly alter the response to H1N1 infection. Understanding the role of As in response to such viral challenges will be important in the overall assessment of the public health risk.

## Materials and Methods

### Animal husbandry

All animal studies were conducted in accordance with guidelines approved by the Association for Assessment and Accreditation of Laboratory Animal Care using a protocol approved by the Institutional Animal Care and Use Committee (IACUC) at Dartmouth Medical School. All animals were treated humanely and with regard for alleviation of suffering. Seven-week-old C57BL/6J male mice (Jackson Laboratories, Bar Harbor, ME) were housed on Carefresh bedding (International Absorbents, Inc., Ferndale, WA) in autoclaved cages and fed AIN-76A diet (Harlan-Teklad, Madison, WI) *ad libitum.* Background As concentrations in the diet were < 20 ppb (Kozul et al. 2008). At the start of the experiment, animals were given drinking water (*ad libitum*; water was changed weekly) with or without the addition of 100 ppb sodium arsenite. Mice were maintained on control or 100-ppb As water throughout the course of infection.

### Influenza virus infection

After 5 weeks of As exposure, mice were anesthetized with 9:1 ketamine:xylazine mix at 0.1 mL/30 g body weight and inoculated intranasally with one-half the median lethal dose of standard laboratory influenza A virus A/PuertoRico/8/34 (H1N1) strain (Flu). Morbidity (measured as weight loss) was monitored daily over the course of infection. Body weight loss > 20% was the end point for termination from the study in compliance with IACUC guidelines.

### Viral titer

Whole lungs from infected mice were homogenized, snap frozen, and stored at − 80°C. We quantified influenza virus titers in whole-lung homogenate using TCID_50_ (50% tissue culture infective dose) determination. Briefly, 10× serial dilutions of lung samples were added in triplicate to Madin-Darby canine kidney (MDCK) cells in a 96-well plate, and plates were incubated at 37°C for 5 days. Infected cells were identified by chicken red blood cell hemagglutination.

### Bronchoalveolar lavage fluid (BALF)

Lungs were lavaged *in situ* with 1 mL phosphate-buffered saline (PBS). Remaining cells were collected with four sequential washes. We centrifuged BALF and combined cells retrieved from all five washes. We used a hemocytometer with trypan blue exclusion to obtain total cell counts (excluding nonnucleated cells). Cytospin preparations were stained using the Protocol Hema 3 stain set (Fisher, Houston, TX). Measurements of cytokine and albumin levels in BALF were assessed from the first 1 mL of BALF only. We used the albumin ELISA kit (Bethyl Labs, Montgomery, TX) to assay albumin concentrations in BALF following the manufacturer’s instructions.

### Mediastinal lymph node (MLN) cell suspensions

We prepared cell suspensions from the MLNs by gentle mincing and digestion in collagenase (1 mg/mL, type II; Worthington Biochemical Corp., Lakewood, NJ) and DNase (0.02 mg/mL, grade II from bovine pancreas; Roche, Manheim, Germany) for 45 min at room temperature, followed by the addition of EDTA (0.1 M) for an additional 5 min.

### Peripheral blood oxygen saturation (SpO_2_)

We measured SpO_2_ using the MouseOx system (Starr Life Sciences Corp., Allison Park, PA) according to the manufacturer’s protocol. Conscious mice were restrained, and the sensor was placed at the base of the tail. Mice were acclimated in the restraint, and data were collected for a period of 10 sec per mouse.

### Flow cytometry

We collected BALF and MLN single-cell suspensions as described above. Red blood cells were lysed by incubation with buffered ammonium chloride solution. FITC (fluorescein isothiocyanate)–conjugated CD4, PerCpCy5.5-conjugated CD8, APC-conjugated CD11c, and PerCpCy5.5-conjugated B220 antibodies were purchased from eBioscience (San Diego, CA). Biotin-conjugated CD103 antibody was purchased from BioLegend (San Diego, CA). Samples were run on FACSCalibur cytometer (BD Biosciences, San Jose, CA), and all data were analyzed with FlowJo software (Tree Star, Ashland, OR).

### Cytokine profile analysis

We analyzed cell-free BALF using the Bio-Plex suspension array system using fluorescently dyed Luminex microspheres beads (Bio-Rad, Hercules, CA). Cytokines and chemokines measured in this study were interleukin-1β (IL-1β), IL-6, macrophage inflammatory protein 1α (MIP-1α), RANTES, monocyte chemoattractant protein 1 (MCP-1), IL-10, macrophage-colony–stimulating factor (M-CSF), macrophage inflammatory protein-2 (MIP-2), macro phage inflammatory protein 1β (MIP-1β), and tumor necrosis factor-α (TNFα). BALF samples were assayed in duplicate. Cytokine/chemokine standards were prepared in PBS and were assayed in triplicate. The assay was performed according to manufacturer protocol.

### DC isolation and culture

The DC culture protocol was described previously (Castellino et al. 2000) and is a modification of that described by Inaba et al. (1993). Briefly, bone marrow cells were collected from the femurs of C57Bl/6J mice exposed to drinking water with or without 100 ppb As for 5 weeks (in the absence of infection). Cells were resuspended at 10^6^ cells/mL in RPMI 1640 medium [10% heat-inactivated fetal bovine serum, 100 U/mL penicillin/streptomycin, 50 mM β-mercaptoethanol, 5% cell culture supernatant from X63 cells secreting granulocyte M-CSF (GM-CSF)]. Cells were plated at 2 mL/well into 12-well plates, and all cells were cultured in the absence of As. On days 2, 4, and 6, the cells were washed, nonadherent cells were removed, and fresh medium was applied. On day 7, cells were scraped and collected for transwell migration assays.

### Transwell migration assay

On day 7, bone marrow cells were counted with trypan blue exclusion using a hemocytometer to assess survival. We observed no differences in cell survival between cells isolated from control and As-exposed mice. Transwell plates (6.5-mm-diameter insert, 8.0-μm pore size, polycarbonate membrane; Corning Costar, Corning, NY) were precoated with 2 μg/mL fibronectin, and 500 μL 10 μM ADP was added to the bottom well; 100,000 cells were added in serum-free medium to the top chamber of a transwell plate and allowed to migrate for 2 hr. After the migration period, we removed any cells remaining on top of the membrane and rinsed the membranes with PBS. Migrated cells were fixed, permeabilized with 0.01% Triton X-100 (Sigma, St. Louis, MO), and stained with crystal violet (Sigma). The membranes were mounted on microscope slides for analysis. We used a Q-Fired cooled CCD camera attached to an Olympus microscope to capture 10 random fields per membrane. Migrated cells were counted with SigmaScan Pro imaging analysis software (Systat Software, Chicago, IL). Counts for all 10 fields were averaged to give a mean cell count for each membrane. All experiments were completed at least three times with *n*= 3 per trial.

### Statistical analysis

Statistical analysis was performed with GraphPad Prism, version 5.0a for Macintosh (GraphPad Software Inc., La Jolla, CA) using a two-tailed unpaired *t*-test with 95% confidence interval and one-way analysis of variance (ANOVA; (*p* < 0.05 was considered significant).

## Results

**Figure 1:**
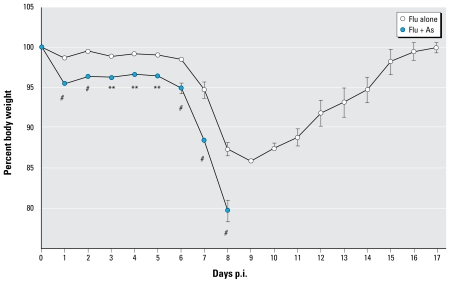
Morbidity of influenza infection (measured by weight loss) in mice exposed to control water or water containing 100 ppb As. One experiment was conducted for days 0–16 (*n* = 6–8 per group), and three additional experimental repeats were conducted for days 0–7 (*n* = 6 per group per experiment). See “Materials and Methods” for experimental details. Values shown are mean ± SEM. The *p*-value for overall significance between groups exposed to flu alone or As + flu is *p* < 0.0001 (two-way ANOVA). **p < 0.01, and ^#^*p* < 0.001, for individual time point exposures.

As shown in Figure 1, after a 5-week exposure to 100 ppb As in drinking water, mice infected with influenza A (H1N1) displayed a significant increase in morbidity. By day 8 postinfection (p.i.), the As-exposed mice displayed such severe morbidity (e.g., body weight decrease ≥ 20%) that those experimental groups were euthanized in compliance with institutional IACUC standards. Because of the severity of these responses, subsequent analyses focused on day 3 and day 7 p.i. In contrast, a parallel group of control mice infected with influenza but not exposed to As displayed a moderate weight loss, but then began to recover weight by day 10 p.i., with complete weight recovery by day 16 p.i. (Figure 1). Exposure to As alone in the absence of viral infection did not influence weight or growth over the 5-week period, nor did anesthesia alone, with or without respiratory exposure vehicle, in either the control or As-exposed mice (data not shown). Thus, the increased morbidity was due to the combination of As in drinking water and influenza infection at an infectious dose at which mice not exposed to As recover.

**Figure 2:**
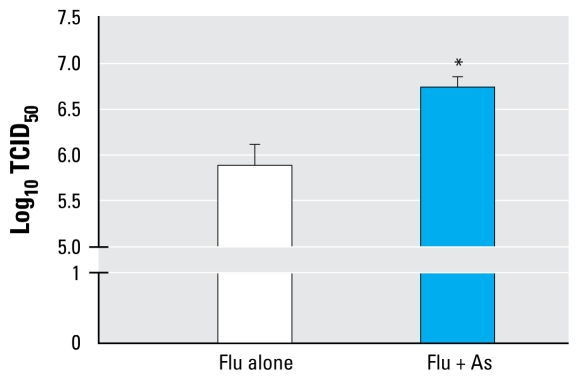
Viral titers for flu on day 7 p.i. in mice exposed to control water or water containing 100 ppb As. Whole-lung homogenates were assessed for viral titers by the TCID_50_ method. See “Materials and Methods” for experimental details. Values shown are mean ± SEM from two experimental repeats (*n*= 3–6 per group). *p < 0.05 by two-tailed Student’s *t*-test.

Given that the inability to properly clear virus from the lung is positively correlated with increased risk of adverse outcomes, we examined the levels of influenza A virus in whole-lung homogenates using TCID_50_ determination at day 7 p.i. Relative to As-unexposed controls, the As-exposed mice exhibited a significant 10-fold increase in viral titers at this time point, correlating with their relative increase in morbidity (Figure 2).

**Figure 3:**
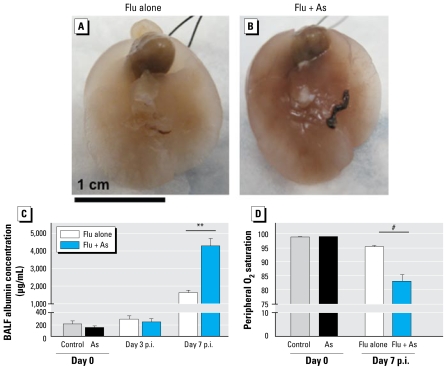
Effect of chronic exposure to As (100 ppb) in drinking water for 5 weeks followed by inoculation with influenza A. (*A, B* ) Gross histology of representative lungs from mice given control drinking water (Flu alone; *A*) and As in drinking water (Flu + As; *B*) at day 7 p.i. after lungs were perfused with PBS and inflated. (*C* ) BALF assessed for albumin by ELISA on day 0 and day 3 and day 7 p.i. (*D* ) Oxygen saturations measured with the MouseOx system at day 0 and day 7 p.i. In *B* and *C*, values are mean ± SEM from one representative experiment (*n*= 6 per group). **p < 0.01, and ^#^*p* < 0.001, by two-tailed Student’s *t*-test.

At day 7 p.i., obvious gross histologic changes in the As-exposed mice, including edema and hemorrhaging, could be observed by visual inspection of the whole lung (Figure 3A,B). We measured capillary leakage into the lungs by assaying albumin concentrations in the BALF. Albumin concentrations were significantly increased in the As-exposed versus unexposed mice at day 7 p.i. Albumin concentrations did not differ between control or As-exposed mice before infection or at day 3 p.i. (Figure 3C). Because an increased severity of influenza infection often correlates with a decrease in SpO_2_, we measured the oxygen saturation levels in infected control and As-exposed conscious mice at day 7 p.i. The As-exposed mice displayed a significant decrease in SpO_2_ levels compared with control mice (Figure 3D). The average SpO_2_ reading for the control mice infected with influenza was 95.2% (range, 93–97.5%), and the average SpO_2_ reading for As-exposed mice infected with influenza was 82.9% (range, 75–90%). Three of the six As-exposed mice had SpO_2_ readings considered to be dangerously low (i.e., ≤ 80%). Exposure to As in the absence of infection had no effect on peripheral oxygen saturation levels.

**Figure 4:**
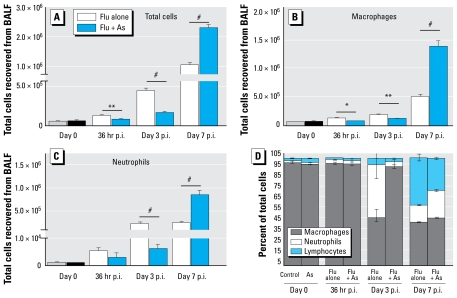
Alteration in cell numbers at day 0, 36 hr, day 3, and day 7 p.i. in BALF of mice exposed to control water or water containing 100 ppb As (Flu + As) followed by inoculation with influenza A. (*A*) Number of viable nucleated cells recovered from BALF. (*B*, *C*) Extrapolation of the total number of macrophages (*B*) and neutrophils (*C*) recovered from BALF. See “Materials and Methods” for experimental details. For A–C, data represent mean ± SEM from one representative experiment (*n* = 5–6 per group). (*D*) Neutrophils, macro phages (Macs), and lymphocytes shown as percentages of the total cells recovered. *p < 0.05, **p < 0.01, and ^#^*p* < 0.001 determined by two-tailed Student’s *t*-test.

We investigated cellular infiltration into the lungs at 36 hr and at day 3 and day 7 p.i. We previously reported that exposure to 100 ppb As for 5 weeks in uninfected mice did not induce changes in the total number of cells recovered from BALF, nor did it alter gross changes in lung histology (Andrew et al. 2007; Kozul et al. 2009). However, in the present experimental model, we observed significant differences in the total number of cells recovered postinfection from the BALF of control and As-exposed mice. At 36 hr and day 3 p.i., As-exposed mice had a significant decrease in the total number of cells recovered from the BALF compared with control mice. Conversely, at day 7 p.i., As-exposed mice had a significant increase in the number of cells in BALF (Figure 4A) relative to controls.

**Figure 5:**
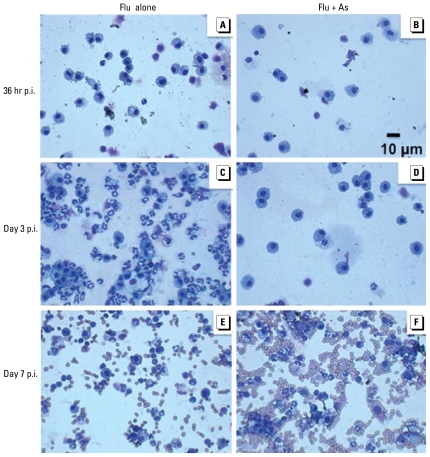
Effects of flu alone and flu plus chronic exposure to As (100 ppb) in drinking water shown at 36 hr, day 3, and day 7 p.i. in representative cytospin preparations. See “Materials and Methods” for experimental details. (*A, C, E*) Flu alone. (*B, D, F*) Flu + As. (*A, B*) 36 hr p.i. (*C, D*) Day 3 p.i. (*E, F* ) Day 7 p.i

To assess whether changes in total cell populations were accompanied by changes in the specific cell types recruited to the lung, we conducted a morphologic analysis of the cells after staining cytospin preparations. Representative cytospin preparations are shown in Figure 5A–F. The total number of macrophages (Figure 4B) and neutrophils (Figure 4C) were significantly decreased at the early stages of infection and increased at day 7 p.i. in As-exposed mice compared with controls, whereas lymphocytes were decreased in the As-exposed mice at day 3 p.i. but were similar to controls at day 7 p.i. (data not shown). In addition to these differences in cell number, the percentages of these different cell types within the total cells recovered from the BALF also changed, as shown in Figure 4D.

**Figure 6:**
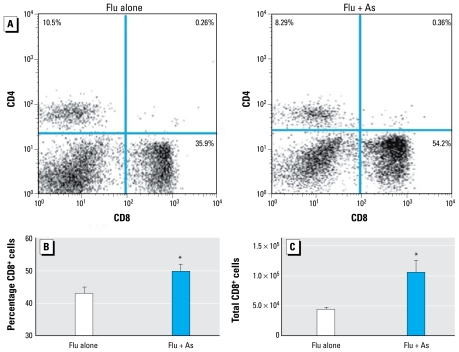
Effects of chronic exposure to As (100 ppb) in drinking water on CD8^+^ cells in BAL in response to influenza A. (*A* ) Representative fluorescence-activated cell sorting plots showing isolated BALF cells stained with fluorochrome-conjugated antibodies against CD4 and CD8. (*B, C* ) Percentage of CD8^+^ cells (*B*) and average number of total CD8^+^ cells (*C*) recovered from BALF at day 7 p.i.; values shown are mean ± SEM from one representative experiment (*n* = 5–6 per group). *p < 0.05, determined by two-tailed Student’s *t*-test.

Although the absolute number of lymphocytes was largely unaffected at day 7 p.i., when subtypes within the lymphocyte populations were analyzed by flow cytometry, CD8^+^ cells were significantly increased in terms of both cell percentages (Figure 6B) and total cell numbers (Figure 6C) in As-exposed mice relative to controls. The percentage of CD4^+^ T cells did not differ by As exposure (data not shown).

**Figure 7:**
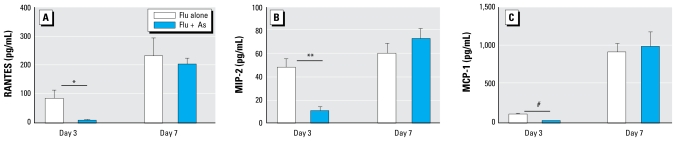
Effects of chronic exposure to As (100 ppb) in drinking water on cytokine and chemokine levels in BALF at day 3 and day 7 p.i. (*A*) RANTES. (*B*) MIP-2. (*C*) MCP-1. See “Materials and Methods” for experimental details. Values shown are mean ± SEM (*n* = 5–6 per group). *p < 0.05, **p < 0.01, and ^#^*p* < 0.001, determined by two-tailed Student’s *t*-test.

We previously reported that As exposure decreased basal cytokine levels in the lungs of uninfected mice (Kozul et al. 2009). In the present study we used a Bio-Plex assay to examine cytokine production in BALF at days 3 and day 7 p.i. We examined a panel of 10 cytokines: IL-1β, IL-6, MIP-1α, RANTES, MCP-1, IL-10, M-CSF, MIP-2, MIP-1β, and TNFα. With the exception of MIP-1α, cytokine production had the same general pattern. We observed no significant differences between the control and As-exposed mice at day 7 p.i. However, we observed a significant decrease in the As-exposed mice relative to controls in 9 of the 10 cytokines at day 3 p.i. (Figure 7). In some cases, such as IL-1β and TNFα, cytokine production was below the limits of assay detection in the As-exposed mice. MIP-1α levels were below detection at day 3 p.i. in all mice but indicated a trending increase in the As-exposed animals relative to controls at day 7 p.i. (data not shown).

**Figure 8:**
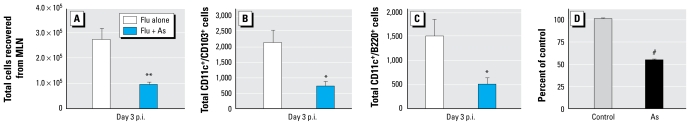
Effect of chronic exposure to As (100 ppb) in drinking water on DCs. See “Materials and Methods” for experimental details. Total number of MLN cells (*A*), CD11c^+^/CD103^+^ cells (*B*), and CD11c^+^/B220^+^ cells (*C*); values shown are mean ± SEM from three representative experiments (*n* = 5 per group). (*D* ) Results of a trans-well assay showing migration capability of DCs toward ADP; Values shown are mean ± SEM from three representative experiments (*n* = 3 per experiment) and are expressed as mean cell migration relative to control mice. *p < 0.05, **p < 0.01, and ^#^*p* < 0.001, determined by two-tailed Student’s *t*-test.

The early recruitment of DCs to the lymph node is essential for the proper initiation of an immune response, and we previously reported that As exposure decreases the expression of genes involved in cell adhesion and migration (Kozul et al. 2009). Therefore, to determine whether the migration of DCs to the lymph node was impaired by As exposure, we analyzed the MLNs for DC populations at day 3 p.i. Single-cell suspensions from MLNs were assessed for the DC markers CD11c^+^/CD103^+^ and CD11c^+^/B220^+^. We observed a significant decrease in the total number of cells recovered from the MLNs in the As-exposed mice relative to controls at day 3 p.i. (Figure 8A). Flow cytometry staining for CD11c^+^/CD103^+^ and CD11c^+^/B220^+^ DCs also indicated that these DC populations were significantly decreased in MLNs of As-exposed mice compared with controls at day 3 p.i. (Figure 8B,C). We observed no differences in the intensity of the staining (data not shown).

We then assessed effects of As on DC migration capacity, using primary bone marrow DCs isolated from control mice and mice exposed to 100 ppb As for 5 weeks (in the absence of influenza infection). Cells were cultured for 7 days in the presence of GM-CSF to produce immature DCs. Relative to controls, cells isolated from As-exposed mice had a significant decrease in migration capability toward ADP in a transwell assay (Figure 8D), indicating that As alone can compromise aspects of immune cell function and that this then manifests as a significantly altered innate immune response after viral infection.

## Discussion

To determine whether chronic low-dose drinking water As exposure compromises the immune response to respiratory infection, we analyzed the outcome of influenza A (H1N1) infection in mice drinking water containing 100 ppb As. Our studies demonstrated that As exposure had a significant impact on the immune system, resulting in a severely compromised response to influenza A infection and increased morbidity. We also observed increased viral titers, capillary leakage, altered cellular responses, and decreased cytokine production and oxygen saturations in the As-exposed mice relative to controls. At early time points postinfection, we observed that As-exposed mice had an attenuated response. Conversely, by the later time points, the As-exposed mice displayed an excessive cellular inflammatory response to infection, indicated by significant increases in the number of cells within the lung and increases in markers of lung injury, such as BALF albumin concentrations and decreased oxygen saturations compared with controls.

We expected the initial attenuated immune response in the As-exposed mice based on our previous results (Kozul et al. 2009). As exposure affects a variety of aspects of host defense, including responses of macrophages (Lemarie et al. 2006), lymphocytes (Andrew et al. 2008; Hernandez-Castro et al. 2009), and airway epithelial cells (Olsen et al. 2008). Additionally, an appropriate and sufficient response to an infectious challenge requires integration of the innate and adaptive immune defenses. Given that As exposure has important impacts on both components, it is not unexpected that the effects of As exposure on the immune response to viral infection are complex. It is therefore likely that several mechanisms contributed to the adverse outcomes observed in the As-exposed mice.

The switch from an inadequate innate immune response at early time points to an excessive response at later time points is intriguing, and the underlying mechanism for this biphasic response warrants future investigation. The excessive response at late time points was also accompanied by considerable hemorrhaging and leakage within the lung. It does not appear that this switch was principally chemokine driven, because we observed that chemokine levels were not increased at day 7 p.i. Moreover, at this time point there were two to three times more cells in the lungs of the As-exposed mice compared with controls, suggesting that cytokine and chemokine production may still be decreased on a per cell basis. However, given that cytokine and chemokine production is a dynamic process, we cannot exclude that an increase in cytokine production may have occurred but was not detected at the time points investigated in this study. It is possible that the increased number of macrophages and neutrophils at day 7 p.i. may have resulted from a compensatory innate response that was not properly initiated early in the course of infection. An overly exuberant macrophage and neutrophil response has also been observed with highly pathogenic strains of influenza, such as H5N1 (Perrone et al. 2008) and the 1918 strain (Kobasa et al. 2007). Nevertheless, the lymphocyte response was ineffective in controlling levels of virus within the lung, because the titers were increased 10-fold in the As-exposed mice relative to control mice at day 7 p.i. The increased viral titers in conjunction with the increase in CD8^+^ cells in the As-exposed mice suggest the possibility that the CD8^+^ cells may not have been fully capable of expressing antiviral effector activities. Alternatively, the impaired innate response may have resulted in unhindered viral replication that even an exaggerated CD8^+^ T-cell response could not effectively control. It is difficult to assess the role of CD8^+^ T cells in the response using this model because of the high morbidity in the As-exposed mice.

We have also shown that As decreased cell migration, which suggests that the critical components of this response were defective. With respect to respiratory viral infection, DC migration to the regional lymph nodes early in the course of infection is a critical component for initiating an appropriate immune defense against influenza infection (Legge and Braciale 2003). The CD11c^+^/B220^+^ DCs in the mouse lymph node are presumptively immature plasmacytoid DCs, which are capable of producing large amounts of type I interferon (Nakano et al. 2001; Siegal et al. 1999). The CD11c^+^/CD103^+^ DCs have been shown to reside in lung mucosa (Sung et al. 2006). The relative decrease in this population of DCs in the lymph nodes may have contributed to the initial attenuated response of As-exposed mice and suggests that antigen presentation within the lymph node from this subset of resident lung DCs may have been compromised. Defective migration by bone-marrow–derived DCs isolated from uninfected As-exposed mice demonstrated that As exposure alone compromised critical functional components of the innate host defense mechanisms, which would likely have affected responses to many pathogens and other pathologic conditions.

The immune response against pathogens is a tightly regulated equilibrium, requiring precise management of effectively clearing pathogen while minimizing immunopathologic damage to the host (Bachmann and Kopf 2002). Therefore, it is not unexpected that the initial impairment in an appropriate immune response due to As exposure could have significant consequences on the ability to clear influenza infection, whereas prolonged viral carriage may predispose to enhanced tissue injury. To our knowledge, this is the first report showing that chronic low-dose As exposure significantly impairs the immune system, and specifically DCs, in response to a respiratory virus infection. Further investigation into mechanisms and the effects at lower doses of As, such as 10 ppb, will be the basis of future studies. We also hypothesize that As exposure will predispose individuals to aberrant responses to other types of infection. In addition to the potential acute effects reported in this study, these aberrant responses may place exposed individuals at risk for development of other chronic lung diseases such as bronchiectasis, which has been reported to be significantly elevated in As-exposed populations (Smith et al. 2006). These results also suggest that chronic As exposure, particularly in areas of Southeast Asia and Mexico, may be a factor that could enhance the potential impact of a pandemic strain of influenza if it should emerge in the human population.
